# Maintenance of Large Subpopulations of Differentiated CD8 T-Cells Two Years after Cytomegalovirus Infection in Gambian Infants

**DOI:** 10.1371/journal.pone.0002905

**Published:** 2008-08-06

**Authors:** David J. C. Miles, Marianne van der Sande, David Jeffries, Steve Kaye, Olubukola Ojuola, Mariama Sanneh, Momodou Cox, Melba S. Palmero, Ebrima S. Touray, Pauline Waight, Sarah Rowland-Jones, Hilton Whittle, Arnaud Marchant

**Affiliations:** 1 MRC Laboratories Gambia, , Banjul, The Gambia; 2 National Institute for Public Health and the Environment (RIVM), Bilthoven, the Netherlands; 3 Jefferiss Trust Laboratory, Imperial College London, London, United Kingdom; 4 Department of Pediatrics, Bronx Lebanon Hospital Center, Bronx, New York, United States of America; 5 Immunisation Department, Health Protection Agency Centre for Infections, London, United Kingdom; 6 Institute for Medical Immunology, Université Libre de Bruxelles, Charleroi, Belgium; Université de Toulouse, France

## Abstract

**Background:**

In a previously published study, we found that large differentiated subpopulations of CD8 T-cells emerged rapidly after CMV infection in young infants and persisted throughout the following year. Here we describe a follow-up study conducted on the same infants to establish whether the differentiated subpopulations continued through the second year post-infection.

**Methodology / Principal Findings:**

CMV-specific cells identified using tetramers remained more activated and differentiated than the overall CD8 population. The large subpopulation of differentiated cytotoxic (CD28^−^CD62L^−^Bcl-2^low^CD95^+^perforin^+^) cells that emerged rapidly after infection remained stable after two years. No similar subpopulation was found in CMV-uninfected infants indicating that two years after infection, CMV remained a major factor in driving CD8 T-cell differentiation. Although markers of activation (CD45R0 and HLA-D) declined throughout the first year, HLA-D expression continued to decline during the second year and CD45R0 expression increased slightly. The age-related increase in IFNγ response observed during the first year continued but was non-significant during the second year, indicating that the rate of functional improvement had slowed substantially.

**Conclusions / Significance:**

The large differentiated subpopulations of CD8 T-cells that had emerged immediately after CMV infection persisted through the second year post-infection, while levels of activation and functional capacity remained fairly constant.

## Introduction

Human cytomegalovirus (CMV) is a persistent herpesvirus that establishes a lifelong but usually subclinical infection [1]. In adults, infection induces large clones of CMV-specific T-cells that are maintained after viraemia subsides and the infection enters the chronic stage [2]. Over 5% of T-cells typically produce IFNγ in response to CMV [3], and individual clones identified by tetramer staining are often much larger [4], [5].

The CD8 T-cell responses of chronically infected adults are characterised by large subpopulations of differentiated cells [6]–[8]. The subpopulations are maintained throughout adulthood, although they expand in the elderly [6], [9] which implies that the differentiated populations remain stable or increase over time.

The relationship between the CD8 T-cells and CMV is evidently complex, as while a strong CD8 T-cell response is unable to prevent viral excretion in healthy individuals [4] or CMV-associated pathology in renal transplant recipients [2], immunity to chronic CMV infection following bone marrow transplant has been re-established by reconstitution of the CD8 T-cell clones specific for CMV [10]. In healthy people, CMV has been linked to influenza vaccine failure in the elderly [11], [12], implying that CD8-mediated control of CMV infection carries a physiological cost at least in the late stages of life.

Most studies have been carried out in high income countries where CMV infection usually occurs in adulthood [1]. Nevertheless, the emergence of large CMV-specific clones similar to those found in adults has been found in young children in the USA [4].

In low income countries, CMV infection is ubiquitous and usually occurs in infancy [1], [13], which offers the opportunity to recruit a prospective cohort to study the interaction of the infant immune system with a viral infection. We recruited a cohort of Gambian infants at birth and monitored them for the onset of CMV infection during the first year of life and evaluated the development of the CD8 T-cell response over two years following infection.

We found that the median age of diagnosis of CMV infection was eight weeks [14], which was considerably lower than in any other cohort studied. The large clones of CMV-specific cells and large subpopulations of differentiated (CD28^−^, CD62L^−^, Bcl-2^low^, CD95^+^, perforin^+^, granzyme A^+^) cells appeared in the same way as in adults or older children, while very few differentiated CD8 T-cells were present in CMV-uninfected infants of the same age. The phenotype of CMV-specific cells was both more differentiated and more activated (CD45R0^+^, HLA-D^+^) than the phenotype of the overall CD8 T-cell population, though levels of activation fell throughout the first year to the extent that by 12 months post-diagnosis, activation in the overall CD8 T-cell population was similar in CMV-infected and uninfected infants of the same age. However, IFNγ production in response to class I restricted CMV peptides was relatively low in the younger infants sampled, indicating that the functional potential of the infants' CD8 T-cells was not as well developed as their phenotypic maturity suggested and was independent of the declining levels of activation [15].

We subsequently sampled a subset of subjects at the end of the second year post-diagnosis, and here we compare the phenotype and function of CD8 T-cells after 12 and 24 months of CMV infection.

## Methods

### Subjects

The initial protocol for the study of the development of the CD8 T-cell response to CMV involved sampling infected infants up to one year after infection and uninfected infants when they reached twelve months of age [15]. It was not logistically possible to follow up the whole cohort for a second year so a subcohort that included infants infected at various ages was identified for sampling at 24 months post-diagnosis, or at 24 months of age among infants that had remained uninfected by 12 months of age. Recruitment of the subcohort was biased towards those infants whose CD8 T-cells bound the available tetramers, and was intended to ensure that infants infected early and late in the first year of life were represented. It consisted of two infants that were infected congenitally, eight infected at 5–9 weeks of age, ten infected at 20–40 weeks of age and two that had not been infected by 24 months of age. The 12 month post-diagnosis sample was collected from infants with ages ranging from 47 to 71 weeks with a mean of 65, while the 24 month post-diagnosis sample was collected from infants with ages ranging from 103 to 144 weeks with a mean of 117.

Infants were recruited at birth from the maternity ward of Sukuta Health Centre. Informed consent was obtained from their mothers and documented by signature or thumb print at birth, and consent was confirmed at the end of the first year following diagnosis. Recruitment was restricted to healthy singleton infants, defined by a birth weight of at least 1.95 kg, without congenital abnormalities. The catchment area of Sukuta Health Centre is peri-urban, and characterised by low income and crowded living conditions. The HIV status of study subjects was unknown, but adult prevalence in the region was 1–4% at the time of the study (National AIDS Control Program, unpublished data) so is unlikely to be a significant confounder of this study.

Limited molecular HLA typing was carried out as previously described [16], [17] on buffy coats obtained from umbilical cord blood separated on a lymphoprep column (Axis-Shield plc, Dundee, Scotland) in order to identify subjects with HLA- A2, B7, B8 and B35 for which known epitope peptides were available.

The study was approved by the Medical Research Council (MRC)-Gambian Government Ethics Committee.

### Sample collection and diagnosis of CMV

A 5 ml venous blood sample was collected into heparin and transported to the laboratory within 4 h. Whole blood was used for flow cytometry, and PBMCs separated on lymphoprep (Axis-Shield) columns for use in ELISpot assay.

In the initial cohort, the onset of CMV infection was diagnosed by the excretion of CMV DNA in the urine, as the infants were young enough for serological testing to be confounded by maternally derived IgG. As maternally derived antibodies would have been cleared by two years of age, the infection status of infants that were not known to be positive was assessed by testing for CMV-specific IgG with an ELICYTOK-G ELISA (Diasorin).

None of the infants showed any CMV-related pathology.

### Flow cytometry

All flow cytometry reagents were from Becton Dickinson. The CD8 population was identified using PerCP-conjugated anti-CD8 antibody. Preliminary experiments demonstrated that it was possible to identify a pure population of CD3^+^CD8^high^ T-cells and that T-cells binding tetramers expressed CD8 at the same high level (unpublished data). Further staining for surface markers was carried out using APC-conjugated antibodies to CD27, CD45R0, CD62L and CD38, and FITC-conjugated antibodies to CD28, CD45RA, CD95 and the class II HLA-D molecules DR, DP and DQ. Intracellular markers were identified using FITC-conjugated antibodies to Bcl-2, Ki-67, perforin and granzyme A. Where possible, cells specific for the immunodominant pp65 protein of CMV [3] were identified with PE-conjugated B7-TPRVGGGAM and A2-NLVPMVATV tetramers, synthesised as previously described [17], [18]. Red blood cells were lysed using FACSlyse solution and lymphocytes were permeabilised using FACSperm II. Samples were acquired on a four-colour FACSCalibur.

### Analysis of flow cytometry data

The flow cytometry analysis of the samples collected during the first year of follow-up were analysed using the automatic processing tool that was developed to analyse the first 12 months of data [15], [19]. Cut-offs and subpopulation statistics from the automatic gating algorithms are written directly to an Access database (Microsoft), and read directly from the database into Stata 9 (Statcorp) for analysis.

The 12 and 24 month post-diagnosis timepoints were compared using a random effects model which allowed for those subjects that had observations at both timepoints. Where necessary responses were appropriately transformed to improve the normality and constant variance regression assumptions. A cross-sectional analysis was used to compare CMV infected with uninfected subjects at 24 months. Interactions between tetramer^+^ and tetramer^−^ populations and follow-up time were tested and if not significant at the 5% level, comparisons were based on the main effects of tetramer and follow-up time.

### ELISpot

A kit for the detection of IFNγ (Mabtech) was used with polyvinylidene difluoride-coated microplates (Millipore) in ELISpot assays. The peptides used were the A2-restricted MLNIPSINV, NLVPMVATV, VLGPISGHV, YILEETSVM and IAGNSAYEYV, the B7-restricted RPHERNGFTV, TPRVTGGGAM and CRVLCCYVL, the B35-restricted IPSINVHHY and VFPTKDVAL, and the B8-restricted DANDIYRIF [20]–[26].

A detectable response was defined as a mean treatment spot count that was more than 10SFU 10^5^ cells^−1^ greater than twice the mean negative control. These criteria have previously been found to provide high specificity while allowing reasonable sensitivity in the detection of peptide-specific responses [15], [27]. Responses were measured as specific spot forming units (SFU), calculated as the difference between the mean treatment spot count and the mean negative control spot count. Data were analysed using Stata 9, and MINITAB 14 (MINITAB inc).

## Results

### Infection with CMV was almost universal by 24 months of age

Of the infants that had been uninfected at twelve months of age, 17 were identified and tested at 24 months and 12 (70%) were seropositive. At twelve months of age, 85% of infants were infected and assuming that the infection rate among the infants that were followed up was representative of all infants uninfected by twelve months, the infection rate at 24 months of age had risen to 96%.

### Relative sizes of activated and CMV-specific subpopulations remained constant in the second year after CMV diagnosis

Cells specific for CMV were identified by tetramer staining, and the frequencies of tetramer-specific cells were compared between 12 and 24 months post-diagnosis using a random effects model that allowed for the incorporation of subjects for whom data for one or other timepoint was missing.

As in the first year after diagnosis, there was considerable variability in the proportions of CD8 T-cells that responded to a single tetramer at the end of the second year, with frequencies ranging from 0.2% to 8.2% of the total CD8 T-cell population. Nevertheless, the relative sizes of the peptide-specific subpopulations within individuals remained similar to the proportions that had been maintained throughout the first two years following diagnosis (data not shown).

### Declining activation among CD8 T-cells during the second year after diagnosis

**Figure 1:**
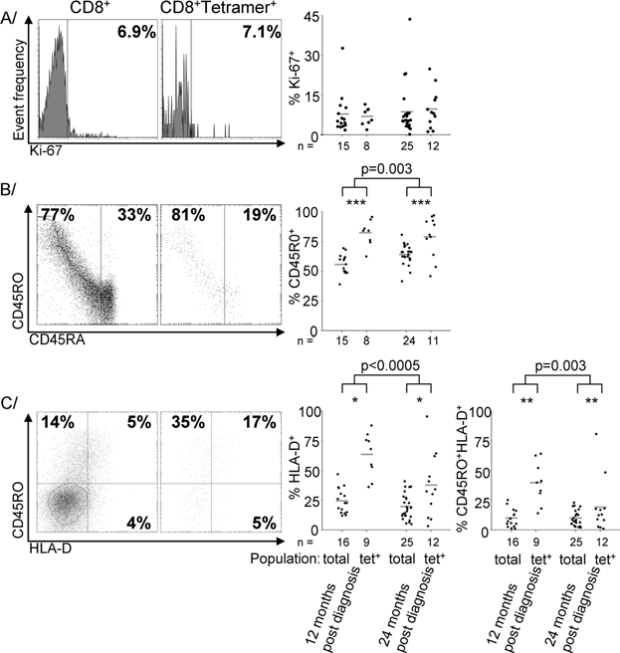
Tetramer^+^ CD8 T-cells are more activated than the overall CD8 T-cell population, but no more so at 24 than 12 months post-diagnosis. A histograms of the expression of Ki-67 in the overall CD8 T-cell population and tetramer^+^ subpopulation of a representative sample collected 24 months post-diagnosis, with individual value plots indicating proportions of Ki-67^+^. B scatter plots of the expression of CD45R0 and CD45RA in the overall CD8 T-cell population and tetramer^+^ subpopulation of a representative sample collected 24 months post-diagnosis, with individual value plots indicating proportions of CD45R0^+^ cells and the CD45 ratio derived from all samples. C scatter plots of the expression of CD45R0 and HLA-D in the overall CD8 T-cell population and tetramer^+^ subpopulation of a representative sample collected 24 months post-diagnosis, with individual value plots indicating proportions of HLA-D^+^ and CD45R0^+^HLA-D^+^ cells and the CD45 ratio derived from all samples. Significances indicate differences between both overall and tetramer^+^ subpopulations. Grey bars indicate means. Significant differences between tetramer^+^ and overall CD8 T-cell populations are indicated by *p<0.05, **p<0.005 or ***p<0.0005.

Markers of activation were identified by flow cytometry and their frequency was compared among tetramer^+^ cells and the overall CD8 T-cell population. The proportion of cells in mitotic cycle, defined by Ki-67 expression [28], was low in both the tetramer^+^ subpopulation and the overall CD8 T-cell population and remained low throughout the second year post-diagnosis (Fig 1A).

Cells stained with CD45RA and CD45R0 were divided into a CD45RA^bright^ subpopulation that did not express CD45R0 and a more activated CD45R0^+^ subpopulation that expressed both isoforms in approximately reciprocal quantities [29], [30]. The CD45R0^+^ subpopulation was more common among tetramer^+^ cells than among the overall population (p<0.0001) (Fig 1B).

Although the proportion of CD45R0^+^ cells had declined through the first year post-diagnosis, it actually increased slightly during the second year among both the tetramer^+^ cells and the overall CD8 T-cell population (Fig 1B).

Activation was further explored by costaining to identify subpopulations that expressed HLA-D, which indicated the secondary expression of class II HLA on activation [31], or coexpressed HLA-D and CD45R0. Tetramer^+^ CD8 T-cells tended to have higher proportions of both the HLA-D^+^ (p = 0.012) and HLA-D^+^CD45R0^+^ (p = 0.001) subpopulations, providing further evidence that they were more activated than the CD8 T-cell population as a whole. Proportions of cells both subpopulations fell during the first year following expression among both the overall and tetramer^+^ CD8 T-cells, but unlike cells expressing CD45R0, the fall continued during the second year of infection among both the HLA-D^+^ (p = 0.021) and HLA-D^+^CD45R0^+^ subpopulations (p = 0.008) (Fig 1C).

### Levels of differentiation remained stable or dropped during the second year post-diagnosis

**Figure 2:**
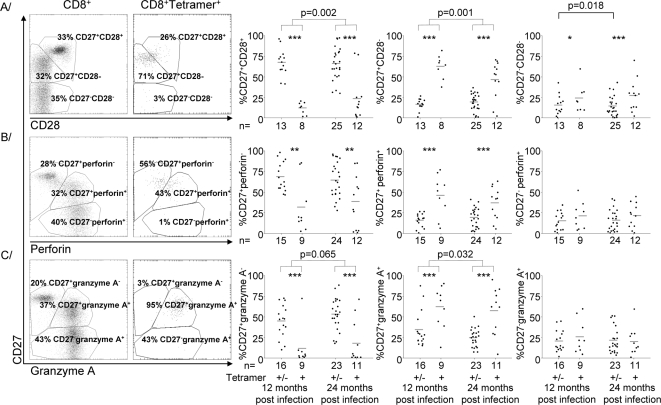
Tetramer^+^ cells are more differentiated than the overall CD8 T-cell population, but there is little change in the relative proportions of the subpopulations in the second year post-diagnosis. A Scatter plots of the expression of CD27 and CD28 gated on all CD8 T-cells and on tetramer^+^ CD8 T-cells, and individual value plots of the percentages of cells that fell into each of the defined subpopulations within the overall CD8 and tetramer^+^ populations. B Scatter plots of the expression of CD27 and perforin gated on all CD8 T-cells and on tetramer^+^ CD8 T-cells, and individual value plots of the percentages of cells that fell into each of the defined subpopulations within the overall CD8 and tetramer^+^ populations. C Scatter plots of the expression of CD27 and granzyme A gated on all CD8 T-cells and on tetramer^+^ CD8 T-cells, and individual value plots of the percentages of cells that fell into each of the defined subpopulations within the overall CD8 and tetramer^+^ populations. Significant differences between sample times are indicated where present. Grey bars indicate means. Significant differences between tetramer^+^ and overall CD8 T-cell populations are indicated by *p<0.05, **p<0.005 or ***p<0.0005.

Differentiation was evaluated by stratifying CD8 T-cells into undifferentiated (CD27^+^CD28^+^), intermediate (CD27^+^CD28^−^) and fully differentiated (CD27^−^CD28^−^) subpopulations [30], [32]–[34]. There were consistently more intermediate and fully differentiated cells among the tetramer^+^ subpopulation than among the overall CD8 T-cell population throughout the second year post-diagnosis (Fig 2A), as there had been throughout the first year.

During the second year post-diagnosis, there were slight but significant increases in the proportions of cells falling into both the undifferentiated (p = 0.002) and intermediate (p = 0.001) subpopulations among both tetramer^+^ cells and the overall CD8 T-cell population. There was a corresponding decrease in the proportion of fully differentiated cells among the total CD8 T-cell population (p = 0.018), which was not mirrored by a significant decrease among tetramer^+^ cells (Fig 2A).

As CD8 T-cells differentiate, they express cytotoxic perforin and granzymes [29], [33], [35]. Consequently, when the stain for CD28 was replaced with a stain for perforin or granzyme A, most tetramer^+^ cells were among the differentiated subpopulations that expressed perforin (Fig 2B) and granzyme A (Fig 2C). Similar trends to those shown by staining of CD28 were evident, although they were not always significant.

**Figure 3:**
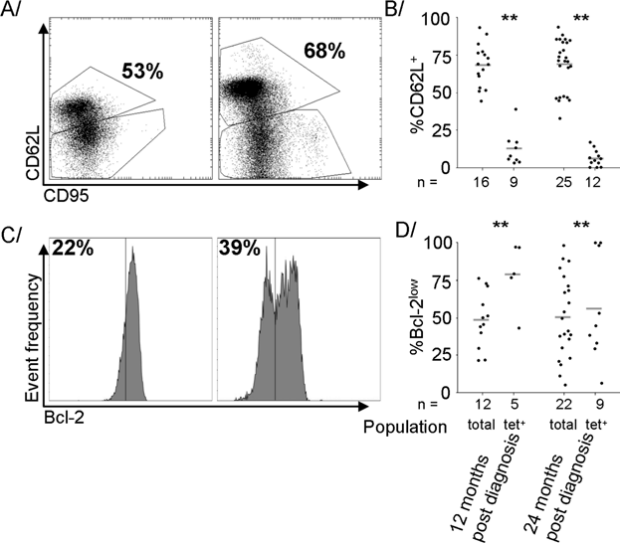
A higher proportion of CD8 T-cells were CD62L^+^ at 24 months than 12, but distribution between the Bcl-2^low^ and Bcl-2^high^ subpopulations remained constant. A Scatter plots of expression of CD62L and CD95 in the overall T-cell population at 12 and 24 months post-diagnosis in a representative sample. B Individual value plots of proportions of CD62L^+^ cells among tetramer^+^ and overall CD8 T-cell populations at 12 and 24 months post-diagnosis. C Histograms of expression of Bcl-2 in the overall T-cell population at 12 and 24 months post-diagnosis in a representative sample. D Individual value plots of proportions of Bcl2^low^ cells among tetramer^+^ and overall CD8 T-cell populations at 12 and 24 months post-diagnosis. Grey bars indicate means. Significant differences between sample times are indicated where present. ** indicates difference between tetramer^+^ cells and overall population significant at p<0.005.

Cells stained for CD62L and CD95 were divided into a relatively undifferentiated CD62L^+^ subpopulation [30], [36] that incorporated cells that expressed various levels of CD95, and a CD62L^−^ subpopulation in which nearly all cells expressed CD95 (Fig 3A). Far more of the tetramer^+^ cells than the total CD8 T-cells had lost expression of CD62L, and the proportions remained relatively constant throughout the first [15] and second (Fig 3B) years post-diagnosis.

Expression of Bcl-2 in the second year post-diagnosis also followed the same trends as it had throughout the first, in that the vast majority of the tetramer^+^ cells fell into the relatively differentiated Bcl-2^low^ subpopulation [37] while the overall CD8 T-cell population was more evenly split between the two, and the relative proportions did not change significantly (Fig 3C, D).

### The CD8 T-cells of CMV-infected infants were more differentiated but no more activated than those of CMV-uninfected infants

At 12 months of age, the vast majority of the CD8 T-cells of CMV-uninfected infants had been among the undifferentiated (CD27^+^, CD28^+^, CD62L^+^, perforin^−^, granzyme A^−^) subpopulations while those of CMV-infected subjects of equivalent age had shown substantial proportions of differentiated cells. The expression of markers of activation such as CD45R0, HLA-D and Ki-67 had been unaffected by CMV status [15]. Very few CMV-uninfected subjects remained by 24 months of age and the CD8 T-cell phenotype was only assessed on two, both sampled at 112 weeks of age. While the low numbers precluded statistical comparisons, the CD8 T-cells of uninfected infants remained considerably less differentiated than those of most of their CMV-infected peers.

**Figure 4:**
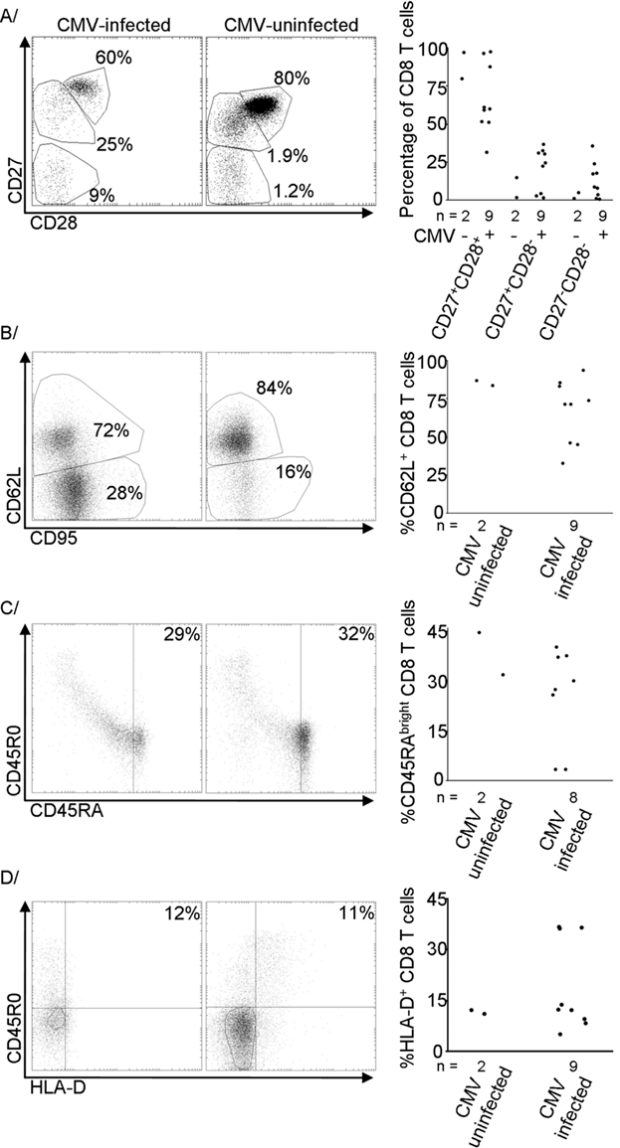
The CD8 T-cells of CMV-infected infants were more differentiated but no more activated than those of CMV-uninfected infants at 24 months of age. A Scatter plots of expression of CD27 and CD28 in representative samples and relative sizes of the three defined subpopulations in all infants sampled at 24 months of age. B Scatter plots of expression of CD62L and CD95 in representative samples and relative sizes of the CD62L^+^ subpopulation in all infants sampled at 24 months of age. C Scatter plots of expression of CD45R0 and CD45RA in representative samples and relative sizes of the CD45RA^bright^CD45R0^−^ subpopulation in all infants sampled at 24 months of age. D Scatter plots of expression of CD45R0 and HLA-D in representative samples and relative sizes of the HLA-D^+^ subpopulation in all infants sampled at 24 months of age. Representative scatter plots are selected as those with the lower proportion of cells in the subpopulation of interest, or the CD27^+^CD28^+^ subpopulation for part A, among CMV-uninfected infants and the median proportion for CMV-infected infants.

At least 80% of the CD8 T-cells of the CMV-uninfected infants fell into the CD27^+^CD28^+^ subpopulation while the mean for the CMV-infected infants was 66% (Fig. 4A), and the lower value for the CD62L^+^ subpopulation among CMV-uninfected infants was 84% while the mean for CMV-infected infants was 67% (Fig. 4B).

Differences of smaller magnitude in the expression of markers of activation were also seen. For instance, the mean size of the CD45RA^bright^ subpopulation was 38% among CMV-uninfected infants and 26% among CMV-infected infants (Fig. 4C) and the mean sizes of the HLA-D subpopulations were 12% in uninfected and 19% in infected (Fig. 4D). While the activated subpopulations were numerically larger in CMV-infected infants in all examples, the standard deviations among the CMV-infected infants were always greater than half of the value of the mean so the differences were considerably less evident than among markers of differentiation.

### Little change in IFNγ responses between 12 and 24 months post-diagnosis

Cells were stimulated with peptides containing known immunodominant epitopes, matched with the appropriate class I HLA restriction. The frequency of IFNγ-producing cells was quantified by ELISpot and compared between 12 and 24 months. Infants with the capacity to respond were identified as those with CD8 T-cells that had recognised one of the two tetramers incorporating the peptide. The proportion that produced a detectable response to the peptide incorporated into the tetramer rose from 16 of 19 (84%) at 12 months post-diagnosis to 11 of 12 (92%) by 24 months.

As there were a number of peptides used in the ELISpot for which tetramers were not available, potential responders were also identified as infants with cells that had produced a detectable IFNγ response to a given peptide in at least one sample. As peptide-specific subpopulations were stable, differences in their detectability were unlikely to be due to fluctuations in their frequency and were considered to represent differences in functional capacity. Based on these criteria, the number of potential responders that actually responded rose from 30 of 68 (44%) at 12 months to 18 of 36 (50%) at 24 months.

While both approaches showed an increase in the proportion of peptide-specific cells that actually responded to the peptide, neither was statistically significant.

## Discussion

We assessed the development of the CD8 T-cell population and its response to CMV after two years of infection, and compared it to the response after one year. The most obvious trend throughout the first year had been for the subpopulations of tetramer^+^ cells to show higher levels of differentiation and activation than the overall CD8 T-cell population [15]. The same large differences remained at 24 months post-diagnosis, as it was evident that almost all CMV-specific cells had lost expression of CD27, CD28 and CD62L and gained expression of CD95, perforin and granzyme A, indicating that they were mature differentiated cells with the potential to kill virus infected cells [32], [35], [38].

The proportion of CMV-specific CD8 T-cells that are highly differentiated (CD27^−^) is much higher than among CD8 T-cells specific for other viruses such as EBV, HIV and HCV [32]. We previously found that the high level of differentiation emerges rapidly after infection, and these data show that it remains for at least two years after infection.

Differences in markers of activation were less striking, although the tetramer^+^ cells expressed elevated levels of CD45RA and HLA-D as they had throughout the first year of infection. The one element of activity that was not upregulated in CMV-specific cells at either 12 or 24 months was division, as defined by expression of Ki-67 [28].

The maintenance of the subpopulations specific for tetramers incorporating the immunodominant pp65 protein indicated no ablation of CMV-specific cells within the CD8 T-cell population, and several individuals retained substantial subpopulations that responded to a single peptide. The long-term maintenance of these populations is in contrast to the reduction in the pp65-specific response over time reported in adults following symptomatic CMV infection [39] or infection during kidney transplant [2]. It is possible that the different patterns of development may be due to the Gambians being infected in infancy or simply that the reduction of the CD8 T-cell response does not occur in asymptomatically infected healthy individuals. Given the huge number of potential epitopes expressed by CMV [3], it is likely that the tetramers were only detecting a small proportion of the overall CMV-specific T-cells and that a substantial proportion of all CD8 T-cells were devoted to the response to CMV.

A substantial subpopulation of all CD8 T-cells had assumed a differentiated, cytotoxic phenotype immediately after diagnosis and the relative size of the subpopulation remained stable throughout the first year of infection. Very few changes were evident 24 months post-diagnosis as there were no changes in the expression of Bcl-2, CD62L or CD95. There was a slight shift of cells from the fully differentiated to the undifferentiated and intermediate subpopulations as defined by CD27 and CD28, indicating that differentiation may not be maintained at the same high levels as in the first year of infection, although the tendency of CD8 T-cells of chronically infected adults to retain a large differentiated subpopulation [6], [7] suggests that the populations of the Gambian infants will never approach their pre-infection levels of differentiation.

At 12 months post-diagnosis, the CD8 T-cell populations of CMV-uninfected individuals contained very few differentiated cells [15] and we concluded that CMV drove differentiation in Gambian children in the same way that has been shown in European adults [8], [40], [41]. Unfortunately with the very low numbers of infants that remained uninfected by 24 months of age, formal statistical analysis is not justified. Nevertheless, the difference in the mean level of differentiated cells between infected and uninfected individuals remained considerable, suggesting that the influence of CMV infection on driving CD8 T-cell differentiation was as important at 24 months of age as it had been at 12.

In contrast to the stability of the differentiated subpopulation, the proportion of cells expressing markers of activation such as CD45R0 and HLA-D fell during the first year post-diagnosis to similar levels to those of CMV-uninfected infants. During the second year, the patterns followed by the two markers diverged as the proportion of HLA-D^+^ cells continued to fall while the proportion of CD45R0^+^ cells actually rose slightly. Similar increases in CD45R0 expression among CD8 T-cells during childhood were observed in the absence of changes in HLA-D expression in a Dutch cohort where CMV infection was probably rare [42], implying that the expression of CD45 isoforms may be more sensitive to the accumulation of antigen exposure during childhood than the expression of HLA-D.

Several studies have shown that the CD8 T-cells of children and infants of equivalent age to those whom we sampled have a weak IFNγ response to viral infections [43]–[45] including CMV [4], making it unlikely that the IFNγ responses of the infants in the subcohort had peaked. However, the slight increase in the second year post-diagnosis, which corresponded to the third year of life for the older children in the sub-cohort, implied that the rate of any increase was considerably less than had been observed in the first year.

In summary, CMV-specific CD8 T-cells remained more activated and differentiated than the overall CD8 T-cell population at the end of the second year of CMV infection. The drop in activation markers among the total CD8 T-cell population that had been clear during the first year following infection was less clear in the second as while the frequency of cells expressing HLA-D continued to drop, the frequency expressing CD45R0 increased slightly. There was a slight but non-significant rise in the proportion of CMV-specific CD8 T-cells that produced IFNγ in response to CMV peptides, indicating that any increase in CD8 T-cell function was considerably less dramatic than it had been during the first two years of life. Nevertheless, the large sizes of peptide-specific clones and continued stability of a large differentiated subpopulation of CD8 T-cells that was absent from uninfected infants indicated that CMV continued to be a major determinant of the state of the CD8 T-cell population.
